# A Citywide Approach to SARS-CoV2 Testing

**DOI:** 10.3389/fpubh.2021.695442

**Published:** 2021-06-30

**Authors:** John P. Broach, Monica Lowell, Olga Brown, Clayton Martin, Michelle Muller, Jeanne Shirshac, Domenica Perrone, Will Smith, Matilde Castiel, Kimiyoshi J. Kobayashi, Cheryl M. Lapriore, Eric W. Dickson, Kavita M. Babu

**Affiliations:** ^1^University of Massachusetts Memorial Health Care, Worcester, MA, United States; ^2^Department of Emergency Medicine, University of Massachusetts Medical School, Worcester, MA, United States; ^3^Department of Health and Human Services, City of Worcester, Worcester, MA, United States

**Keywords:** COVID-19, SARS-CoV2, pandemic, testing, public health

## Abstract

The COVID-19 pandemic caused more than 30 million infections in the United States between March 2020 and April 2021. In response to systemic disparities in SARS-CoV2 testing and COVID-19 infections, health systems, city leaders and community stakeholders in Worcester, Massachusetts created a citywide Equity Task Force with a specific goal of making low-barrier testing available to individuals throughout our community. Within months, the state of Massachusetts announced the Stop the Spread campaign, a state-funded testing venture. With this funding, and through our community-based approach, our team tested more than 48,363 individuals between August 3, 2020 and February 28, 2021. Through multiple PDSA (Plan-Do-Study-Act) cycles, we optimized our process to test close to 300 individuals per hour. Our positivity rate ranged from 1.5% with our initial testing events to a high of 13.4% on January 6, 2021. During the challenges of providing traditional inpatient and ambulatory care during the pandemic, our health system, city leadership, and community advocacy groups united to broaden the scope of care to include widespread, population-based SARS-CoV2 testing. We anticipate that the lessons learned in conducting this testing campaign can be applied to further surges of SARS-CoV2, international environments, and future respiratory disease pandemics.

## Introduction

The COVID-19 pandemic caused more than 30 million infections in the United States between March 2020 and April 2021 (1). Widespread testing remains one of the pillars of the COVID-19 pandemic public health response, as the probability of transmission can be limited by testing, quarantine and isolation (2–4). Testing is especially important given the risk of transmission during the pre-symptomatic period (5), and the need for contact tracing (4). In the early months of the COVID-19 pandemic, testing availability in the United States was limited by availability of test kits, insurance/financial considerations, and the need for symptoms and access to a health care provider to obtain an order for testing. This set of barriers created a significant lack of equity in access to testing; even egalitarian testing resources undersampled lower socioeconomic status populations at highest risk for COVID-19 infection (6).

In response to systemic disparities in SARS-CoV2 testing and COVID-19 infections, health systems, city leaders and community stakeholders in Worcester, Massachusetts created a citywide Equity Task Force with a specific goal of making low-barrier testing available to individuals throughout our community. This task force, co-chaired by the University of Massachusetts (UMass) Memorial Health Care system and the City of Worcester Department of Health and Human Services, was comprised of more than 22 organizations and over 50 individuals.

Our city is located in Central Massachusetts, with a population of 191,575 people (7). In greater Worcester, 21% of the population is of Latino or Hispanic origin, and 13% of the population is Black or African-American. Thirty-four percent of Worcester adults speak another language besides English, with 15% of Worcester residents speaking Spanish (8).

Faced with the important challenge of providing testing to our diverse population of residents, our group began meeting in May 2020, with working groups to address (1) analytics regarding COVID-19 infection data analysis to identify local disease activity; (2) testing strategies; (3) education and outreach, and (4) equity. Each team met individually and together to review data on where outbreaks were occurring, populations at-risk, optimal testing approaches, preventive strategies, and barriers to access.

Within months, the state of Massachusetts announced the Stop the Spread campaign, a state-funded testing venture. With this funding, and through our community-based approach, our team tested more than 48,363 individuals between August, 3, 2020 and February 28, 2021.

Below, we describe the optimization of our community-based strategy to allow for low-barrier community access. We are hopeful that the advent of vaccination programs will slow the transmission of SARS-CoV2 and make the need for testing on this scale less acute. However, we predict that this low-barrier approach will be very helpful for the implementation of similar testing for other upper respiratory infection epidemics. We also are able to refocus our current process on vaccine distribution. And we further anticipate that this information will be useful in the historical perspective on the wide variety of public health responses to COVID-19.

## Context

Through geospatial mapping analysis, our analytics team identified specific census tracts with the highest recent number of COVID-19 cases. Led by the Vice President of Health Policy and Public programs at UMass Memorial Medical Center (JS), this office produced reports of infections in Worcester and surrounding communities on a weekly basis with further breakdown by age, sex/gender, race/ethnicity, and census tract using data from the state of Massachusetts' MAVEN system (integrated case tracking system of record). We additionally obtained information from hospitalized patients, as well as available data from community testing. Based on these analyses, we identified several census tracts with the highest rates of infection in our community. Additionally, we identified the impact of COVID-19 in communities of color. For example, Hispanic/Latinx individuals made up 37% of persons with COVID-19 infections despite making up 21% of the population in February 2021. As such, any testing programs or prevention/ outreach efforts would necessarily be provided in English and Spanish. Of note, the census tracts with the highest rates of COVID-19 infection ranked within the top 10% of communities based on the CDC Social Vulnerability Index; two of these census tracts are within the top 1% using this measure (9).

## Programmatic Details

**Figure 1:**
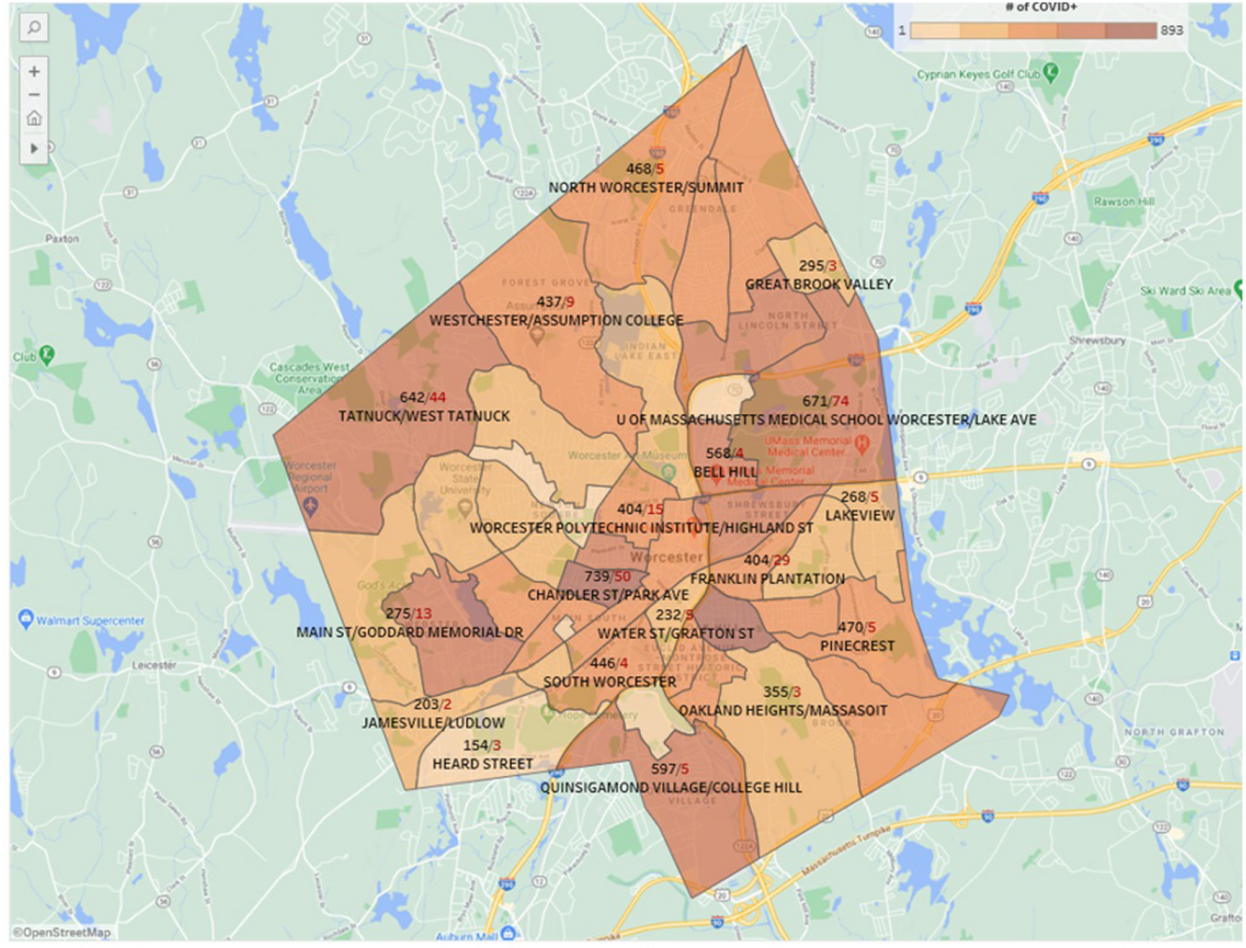
Representative geospatial mapping of SARS-CoV2 positive cases in Worcester, MA from February, 2021. Black numbers represent infections, while red numbers denote deaths.

Utilizing the geospatial mapping, we were able to hone in on the census tracts with the highest number of COVID-19 infections at a street level view within census tracts (see Figure 1). We subsequently identified potential testing venues in high-traffic areas in each target census tract. Sites included churches, the grounds of city hall, local schools, large housing developments, and a community development center. Task force leaders traveled to each potential site, meeting with community stakeholders to discuss suitability and acceptance. We based decisions regarding suitability in part on accessibility to pedestrians and those using public transportation to mitigate this barrier. We selected sites based on accessibility of each site to facilitate walk-up (no appointment necessary) testing, distinguishing our approach from many public testing initiatives. Through outreach into BIPOC neighborhoods alongside community organizations, we were able to identify sites that promoted culturally appropriate services, fostered a sense of trust within their existing networks, and ultimately provided safe, accessible, and comfortable physical environments.

**Figure 2:**
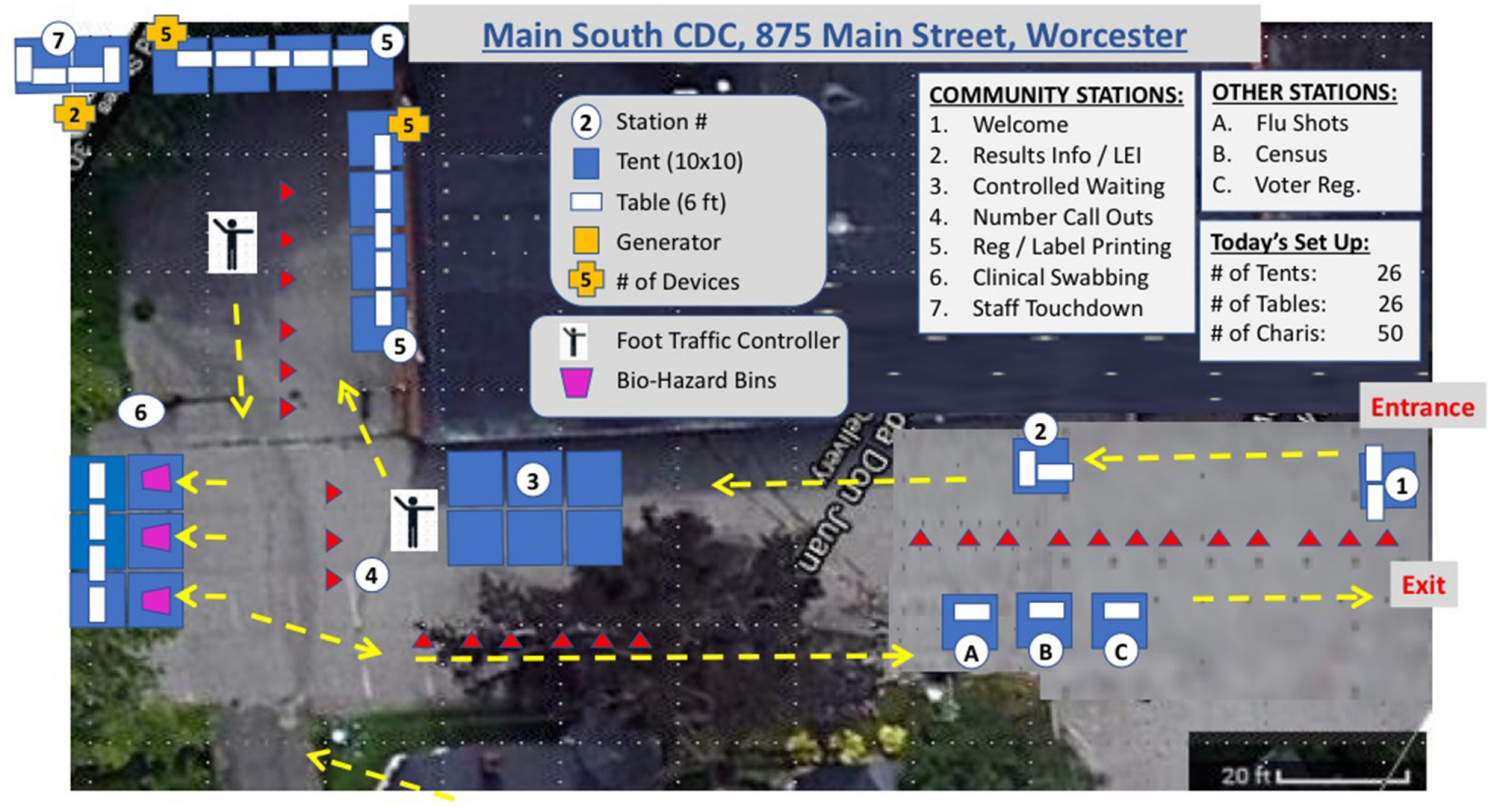
Representative site map (Main South, Community Development Corporation) from August, 2020. LEI, Latino Education Institute.

Prior to each testing date, project managers from the Center for Innovation and Transformational Change provided a site map (please see Figure 2). These site maps traced patient movement through either parking or a walk-up entrance (depending on the site), registration, swabbing, then public health/ public advocacy opportunities. A cellular engineer visited each site in advance to ensure that adequate bandwidth would be available to the Wi-Fi set-ups at each site. Dates and times of testing were disseminated via the local newspaper and multiple social media channels in English and Spanish to optimize engagement by community members. To address the digital divide, we additionally shared the location and timing information by newspaper, through community ambassadors, and via key stakeholders.

There were no requirements for testing with respect to either provider orders or symptoms. A blanket standing physician order allowed testing for all presenting patients. Infants, children of all ages and adults were tested at each site.

## Testing Procedure

During the pandemic, the Ronald McDonald Care Mobile team that usually provides community outreach and dental care to underserved populations was redeployed from that primary mission due to school closures and infection control concerns. As a result, this team turned its operation into “Feet on the Street” in the earliest days of the pandemic, providing education and prevention information and materials in six different languages (33 languages available on our website), as well as hand sanitizer and masks. Once we began these community-wide testing initiatives, the Care Mobile team provided critical personnel, community recognition and trust, and clinical skills to anchor the mobile testing events.

Beyond the Care Mobile team, several groups of personnel were required for the deployment of each pop-up site. A facilities team provided person-power, transportation and equipment to set up tents, set up the wireless system and generator, and allot required PPE to each station. Registration personnel gathered demographic and contact information. Clinicians (typically nursing assistants, nurses, advanced practice providers, and/or physician staff) swabbed patients as they presented for testing. Additional staff members directed patients through the stations, provided language assistance and troubleshot problems as they arose. Registration personnel were hired or redeployed for this effort; volunteers from multiple community partners (including the Latino Education Institute) further supported the registration team. The clinician team was comprised of individuals redeployed for this effort, supplemented by physician and advanced practice provider volunteers, drawn largely from emergency medicine and primary care specialties.

All personnel were required to wear an N95 mask (or equivalent), as well as a surgical mask and eye protection. Clinical staff additionally wore gowns during swabbing; personnel protective equipment was provided on-site to staff.

The registration process was the most high-stakes, complex and time-consuming portion of our process. Data collected included name, date of birth, phone, email (when available), address, race, and ethnicity. Accurate data entry was critical to prevent difficulties reaching patients with results. Data were entered into the electronic platform used by receiving laboratory (Broad Institute/ELLKAY CareEvolve, Cambridge, MA). Each registrar required a laptop computer, power supply, cellular “air card” and wireless label printer. After entering all necessary demographic information, the registrar printed a patient-specific label and applied it to a specimen collection tube. Multiple bilingual staff (English/Spanish, English/Portuguese) facilitated this process. No identification or insurance information was required to access testing. The patient was then directed to a swabbing station.

We chose clinician-administered mid-turbinate swabs based on efficiency. Using this approach, patients were asked to clear (blow) their noses prior to testing. After confirming patient information, clinicians would then swab the patient's anterior nares using a cotton-tipped swab for approximately 15 s on each side. The swabs were then placed in the previously labeled tubes. Samples were transported in batch to the Broad Institute by courier at the end of each testing session.

**Figure 3:**
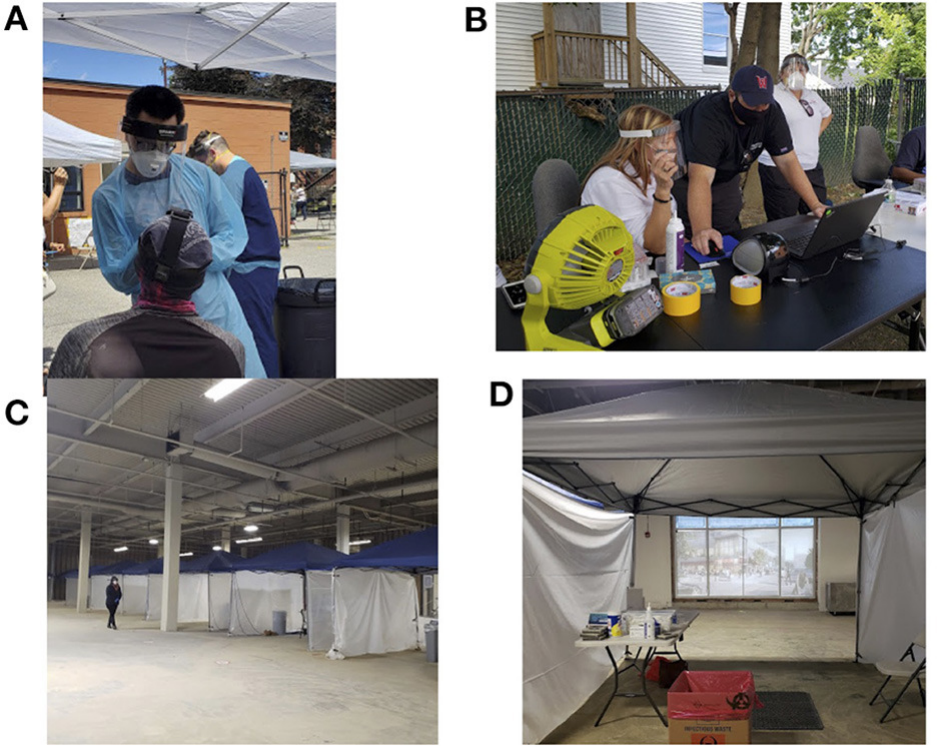
Photos of indoor and outdoor testing set-ups. **(A)** Clinician wearing PPE, swabbing walk-up community member; **(B)** Setting up outdoor registration area; **(C)** Indoor testing venue; **(D)** Indoor testing booth.

Several testing dates were canceled in October due to adverse weather. In November 2020, we moved our testing venue to an indoor location to prevent disruptions due to weather. A large commercial space was donated for this purpose. The location was ideal, located <10 walking minutes from Union Station, Worcester's transportation hub. Additionally, this testing center was located in a socially vulnerable neighborhood with high COVID-19 positivity. The tents used for the pop-up sites were deployed in this 22,000 square foot space, partitioning registration and swabbing staff (see Figure 3 for pictures of the indoor and outdoor testing sites). Fit testing for N95 mask use was required prior to staff participation in these events. To further facilitate this requirement, some staff members were trained to perform fit testing on site for any volunteers or employees who had gone more than 1 year since formal fit testing.

The state-funded testing conferred two critical benefits. First, the turnaround time for testing was approximately 24 to 48 h; second, all negative tests were reported directly to the patient by email, obviating substantial callback burden. Team members shared the responsibility for callbacks to patients with positive results and appropriate counseling. We also created a small call center to provide additional assistance with calling back individuals who tested positive, and to respond to public inquiries regarding testing and results. We contacted all individuals with their results and were able to encourage household contacts to be tested during one of our subsequent testing sessions. All positive SARS-CoV2 tests in MA were and are automatically reported to the MA Department of Public Health because COVID-19 is classified as a disease of high public health consequence. The state team separately followed-up with individuals who tested SARS-CoV2 positive to conduct contact tracing.

Additional resources were provided through the City of Worcester Division of Health and Human Services to address food insecurity for individuals requiring quarantine or isolation. The Department of Health and Human Services referred a total of 204 individuals from the months of August–November to the City's Hot Meal Program, which was coordinated by the Family Resource Center at YOU, Inc. These individuals received hot meal delivery from local restaurants for 2 weeks after their referral.

## Prevention Education and Outreach

Beyond community COVID-19 surveillance, the testing sites afforded an opportunity provide patients with other secondary benefits. Throughout our testing sessions, our team provided education and resources regarding COVID-19 infection prevention. Through outside grant funding, we were able to initially purchase thousands of masks and containers of hand sanitizer. Each individual presenting for testing (or their family members) received a packet containing: two surgical masks; a travel-sized container of hand sanitizer; instructions on avoiding COVID-19 infection and symptom recognition. On site, we partnered with community organizations, such as the Southeast Asian Coalition (SEAC), to provide PPE and other resources. Additionally, we worked with other private organizations and community partners to provide necessary services to our patients. A national retail pharmacy provided staff for the administration of influenza vaccines; over 1,100 vaccines were administered during sessions held in August 10 and September 17, 2020. Other groups, such as Worcester Interfaith, were also on-site to promote PPE and register community members for the United States Census. Through partnership with the League of Women Voters, we also had voter registration on site for several events. Community partner involvement was coordinated by the Department of Health and Human Services for the City of Worcester.

## Results

Between August 3, 2020 and February 28, 2021, our team performed 48,363 tests in community-based, non-medical locations. Our first nine-hour testing event was held on the evening of August 3, 2020, at the Community Development Corporation. We tested 680 individuals, at a rate of 75 patients per hour. Occasionally, tests could not be processed due to issues during the collection, transport, or analysis. During this first testing session, our “not processed” rate was 5%.

**Figure 4:**
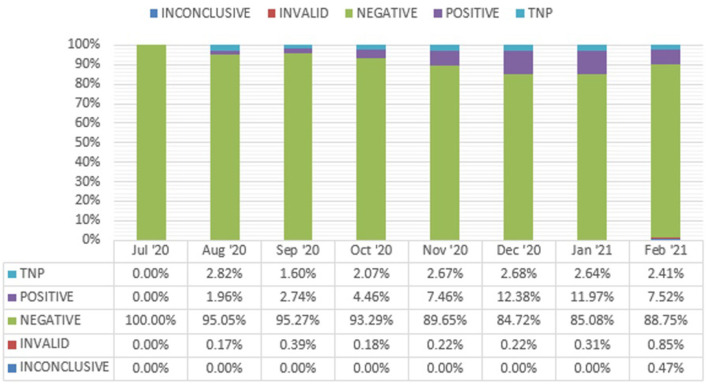
SARS-CoV2 testing result percentages over time. TNP, test not processed.

After this first pop-up testing event, our team made adjustments to subsequent site maps to facilitate testing (and minimal waiting) for individuals with decreased mobility. Our testing sessions averaged four hours in length. Through multiple PDSA (Plan-Do-Study-Act) cycles, we optimized our process to test close to 300 individuals per hour. We did try a patient-administered swabbing approach but found that it did not improve throughput. Additionally, with staff attention to specimen collection, specimen labeling and transportation, our “test not processed” rate rapidly fell to under 1% (see Figure 4).

**Figure 5:**
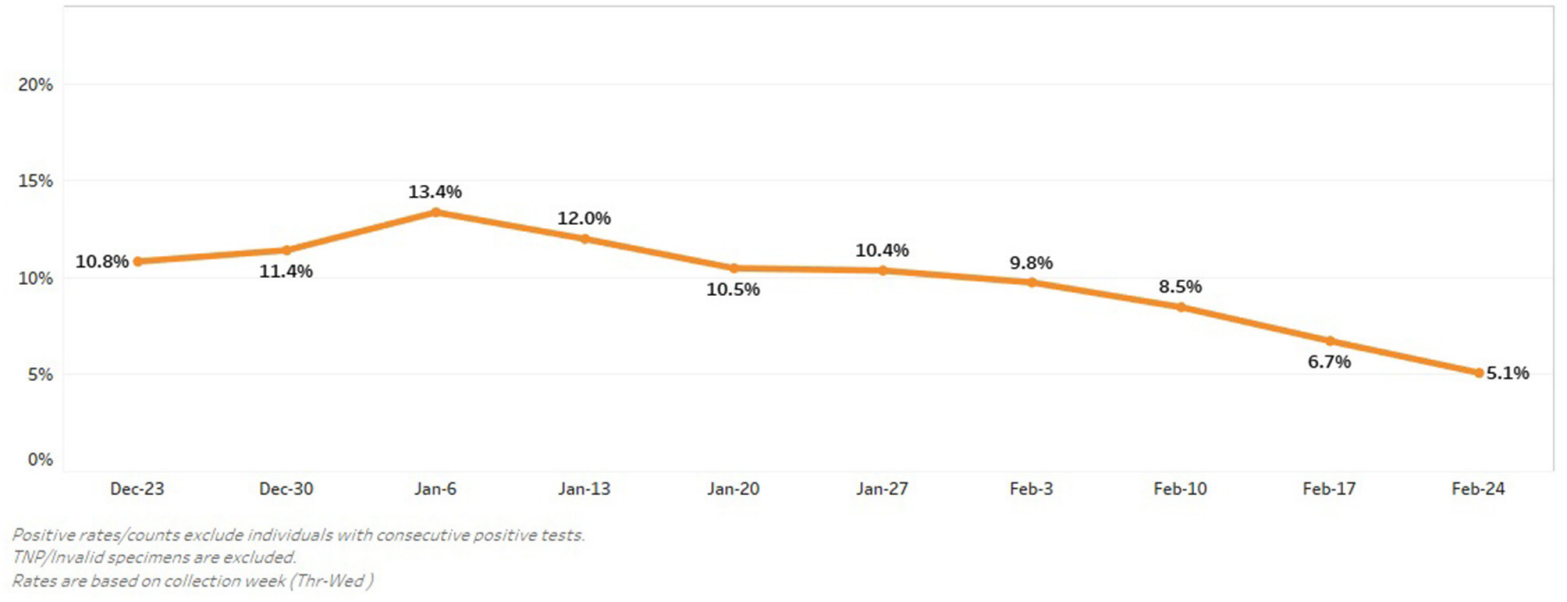
Percent SARS-CoV2 positive tests over time.

From July 2020 to October 2020 (before cold weather led to relocation indoors), we conducted over 25 events in nine locations. We tested over 11,000 people and identified 342 positive community members within the City of Worcester during a window between the first and second COVID-19 infection surges in our community. Our positivity rate ranged from 1.5% with our initial testing events to a high of 13.4% on January 6, 2021 (see Figure 5). Engagement with community members also improved, leading to the testing of 1,388 patients in 4-h testing session on December 28, 2020.

## Discussion

The COVID-19 pandemic in our community led our team to rethink traditional models of public health strategy and delivery. Community public health interventions are usually the purview of cities, municipalities, community health centers and other government-funded organizations. The flagship health care system in our region, UMass Memorial Health Care, stepped in to drive and support this public testing initiative in Worcester, MA in support of a statewide and state-funded testing initiative made further possible by mass viral testing strategies of the Broad Institute.

Although the Care Mobile team functioned as an anchor, event staffing relied on the generosity of volunteers. These volunteers came from UMass Memorial Medical Center (Emergency and other physicians across our system, nurses, administrative assistants, executives, our Worcester EMS, CITC team, and many others), as well as from our community partners (e.g., City of Worcester, Latino Education Institute at Worcester State University, Worcester Interfaith and other organizations). Running the volunteer model required agility on the part of our core team—managing volunteer sign ups, adjusting to and training new volunteers at each event, and executing the testing under conditions of volunteer shortage. Our staff's agility allowed us to handle unexpected challenges, including high winds, changing foot traffic patterns, and community members' individual needs (e.g., mobility, apprehension).

Limitations of this work included lack of a comparison group to document the effectiveness of our intervention; further state-level data could provide additional insight comparing cities with and without Stop the Spread efforts. Additionally, we focused this review on process interventions to increase testing efficiency; we did not assess the role of communications through key community groups, traditional and digital media in creating awareness of our testing service and subsequent impact on volume.

During the challenges of providing traditional inpatient and ambulatory care during the pandemic, our health system, city leadership, and community advocacy groups united to broaden the scope of care to include widespread, population-based SARS-CoV2 testing. We anticipate that the lessons learned in conducting this testing campaign can be applied to further surges of SARS-CoV2, international environments, and future respiratory disease pandemics.

## Data Availability Statement

The datasets presented in this article are not readily available because our data are part of the larger MA Stop the Spread testing effort. We cannot share raw data but can offer aggregate statistics for our site. Requests to access the datasets should be directed to Olga Brown, olga.brown@umassmemorial.org.

## Ethics Statement

Ethical review and approval was not required for the study on human participants in accordance with the local legislation and institutional requirements. Written informed consent from the participants' legal guardian/next of kin was not required to participate in this study in accordance with the national legislation and the institutional requirements.

## Author Contributions

Material preparation, data collection and analysis were performed by OB, WS, CM, and KB. The first draft of the manuscript was written by ML, OB, JB, and KB. All authors contributed substantially to the design, implementation and analysis of this work, commented on previous versions of the manuscript, read, and approved the final manuscript.

## Conflict of Interest

The authors declare that the research was conducted in the absence of any commercial or financial relationships that could be construed as a potential conflict of interest.
